# Staying ahead of the curve: a decade of preprints in biology

**DOI:** 10.1242/bio.062111

**Published:** 2025-06-27

**Authors:** Reinier Prosée, Katherine Brown

**Affiliations:** ^1^ preLights Community Manager, The Company of Biologists; ^2^ Publishing Director, The Company of Biologists

When Cold Spring Harbor Laboratory announced the launch of *bioRxiv* in November 2013, only the braver or more radical amongst us would have predicted that it heralded a lasting change within the science publishing ecosystem. What started with a bold idea **–** an open access preprint repository for the biological sciences (see original news post) **–** resulted in a cultural change within the field, changing the way research is shared, reviewed and accessed. The emergence and expansion of preprint review platforms, funder policy changes and community advocacy for preprint adoption continues to support this change.

The Company of Biologists was one of the earlier advocates among publishers for the integration of preprints into existing publishing workflows. Furthermore, through initiatives such as preLights, the Company pro-actively helps to amplify the reach and impact of preprints. This Editorial explores the transformation of the preprint landscape since 2013 and how the Company has adapted to and embraced this (new) way of sharing research.

## Laying the foundations for preprint adoption

Whereas preprinting in the physical sciences has a much longer history (Tennant et al., 2018), the adoption of preprints in biology took a significant step forward in 2013. After a few unsuccessful attempts to start a preprint server that would encompass the life sciences (e.g. *Nature Precedings* in 2007), the successful launch of *bioRxiv* meant that life scientists now had a dedicated platform to rapidly share their findings (Sever et al., 2019a,b preprint). Of note, other preprint servers (e.g. *PeerJ* Preprints) or related platforms (e.g. F1000Research) were launched around the same time, offering researchers more options to share their work. The Center for Open Science launched the Open Science Framework (OSF) Preprints platform in 2016 to link preprint servers that were starting to appear across the scientific landscape (Tennant et al., 2018). *bioRxiv* quickly became the most popular preprint server for the biological sciences and only recently received competition from Research Square (the preprint server owned by Springer Nature) with regard to this title (see recent statistics collated by Europe PMC).

When, in 2015, Ron Vale noted that it took University of California San Francisco (UCSF) graduates longer and longer to achieve a first-author publication, this inspired him to found the preprint-focused organisation titled Accelerating Science and Publication in Biology, better known as ASAPbio. This non-profit organisation has been instrumental in pushing the wider acceptance of preprints within the biological sciences. Today, ASAPbio continues to play a pivotal role in the preprint ecosystem, advocating for widespread changes (involving the adoption of preprints) that increase accessibility, inclusion and fairness.

Prior to the launch of *bioRxiv*, The Company of Biologists journals' policies were incompatible with preprint posting. However, the Editors-in-Chief and Board of Directors quickly realised that *bioRxiv*, unlike previous attempts, was likely to gain traction – at least in some of our communities. In early 2014, Development and Biology Open began to welcome the submission of preprinted manuscripts. By the end of 2016, not only had all our journals introduced this policy, but we had also integrated with *bioRxiv* to enable bi-directional manuscript transfer between the two sites. It is noteworthy that different areas of the life sciences have embraced preprinting to different extents – with cell and developmental biologists being much more likely to post preprints than comparative physiologists (Nelson and Marshall, 2025). Adoption of preprint-related policies has therefore reflected the needs and wishes of the communities our journals serve.

## Emergence of preprint review platforms

In line with the policy changes at The Company of Biologists, nearly half of publishers had developed preprint-related policies by 2017 (Teixeira da Silva and Dobránszki, 2019). At this point, it was also becoming clear that researchers were increasingly finding *bioRxiv* (and other preprint servers) an invaluable resource for accessing the latest scientific findings. In response, our community site the Node (which serves the developmental biology field) began to collate monthly lists of relevant preprints – making it easier for researchers to find the content they were interested in. From an initial of just 20 preprints in June 2016, the monthly list now typically includes over 150 articles, and these posts on the Node are consistently among its most-read content. The popularity of these posts prompted the Company to consider other ways of helping the community to access and digest the preprint literature, leading to the birth of preLights – a preprint highlighting service that allows early-career researchers (ECRs) to highlight and discuss preprints that spark interest or debate in the scientific community (Brown and Pourquié, 2018; for more information about preLights, see Box 1).

Box 1. preLights (2018 to now) Designed as a community-driven platform, supported by The Company of Biologists and a dedicated Community Manager, preLights allows early-career researchers (ECRs) to highlight and discuss preprints that are of interest to them as well as the wider biological community (Brown and Pourquié, 2018). A usual preLights post contains a summary of the key findings presented in the selected preprint, the reasons it was selected and the preLighter's thoughts on its significance. Inspired by a published study supported directly by the preLights community (Brierley et al., 2022), ‘postLights’ is a recently added feature that tracks how an article changed between the preprinted and published version. In addition, two-thirds of the >1600 preLights posts include a response from the preprint authors, making preLights a unique platform for encouraging discussion around preprints. This also is the main aim of its associated podcast series, ‘spotLights’. preLights is indexed on EuropePMC, *bioRxiv*, PubPeer and Sciety, and all posts receive a DOI. As such, it not only promotes the visibility of preprints but also supports ECRs in building their expertise, writing skills and networks.

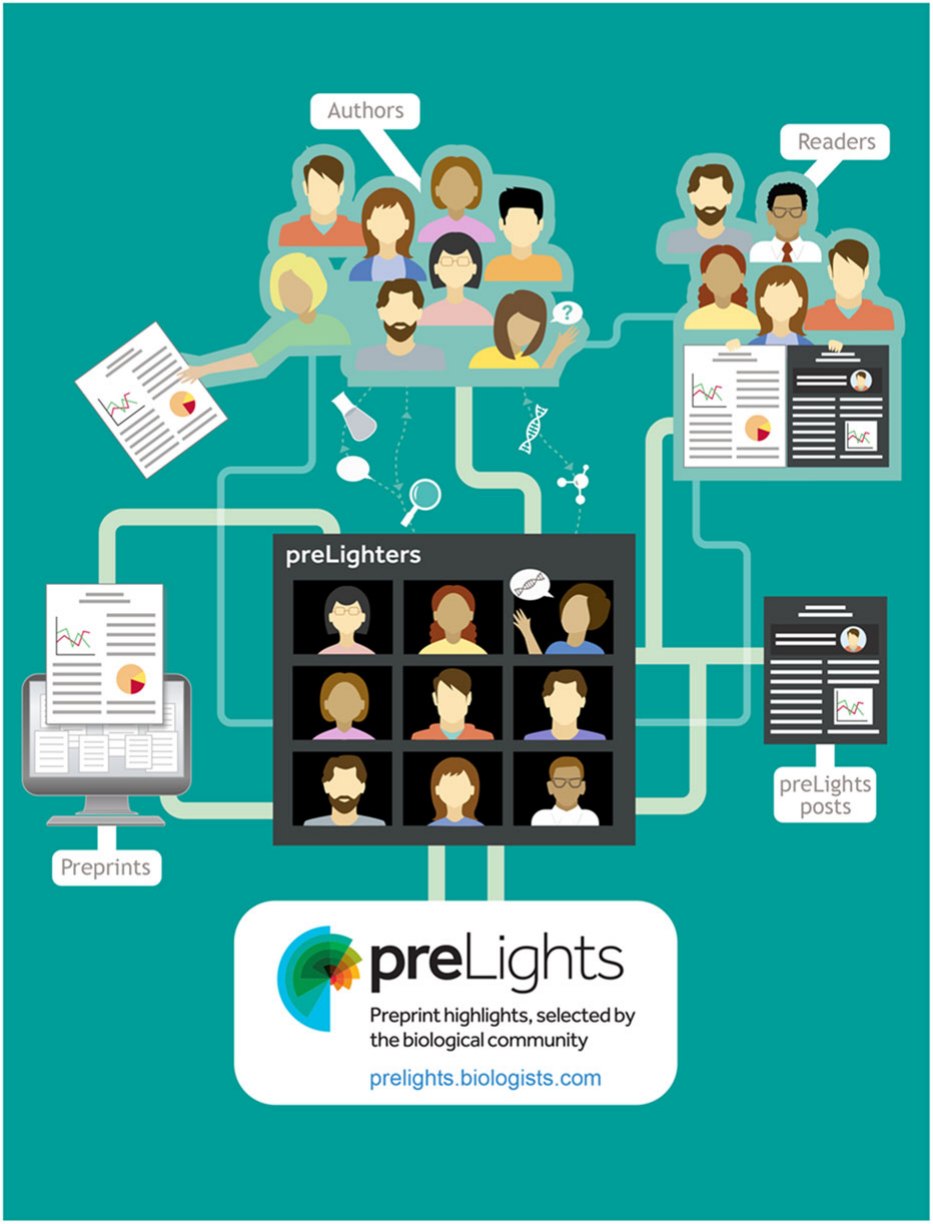

We weren't the only ones to realise that there was a need for collation and review of preprints; around the same time, several preprint review initiatives started to emerge – most notably PREreview and Peer Community In. These sites provide more formal peer review of preprints than preLights but – like preLights – there is a strong focus on involving ECRs and diversifying the pool of researchers that can contribute to the review of the scientific literature. Thus, a new community began to coalesce, led by ASAPbio, around the preprint ecosystem – involving researchers, publishers, funders and other relevant parties.

Besides preprint review platforms, there was also a noticeable increase in so-called overlay journals. These journals combine the available preprint infrastructure (e.g. *bioRxiv*) with existing peer-review policies and journal structures (Rousi and Laakso, 2024). Within the biological sciences, a particularly prominent example of such a journal is *JMIRx | Bio*, which, rather than an overlay journal, defines itself as a ‘Superjournal’. Although journals like *JMIRx | Bio* could, in theory, bridge preprints and publishing workflow, it is interesting to note that, in practice, overlay journals have not gained significant traction to date.

## Consolidating the use of preprints and strengthening the ecosystem

By 2018, the number of life science preprints was growing at a rate ten times faster than that for traditional journal articles (Levchenko et al., 2024). However, journal policies regarding preprints varied widely, and it was not always straightforward for authors to find out what they could do in the preprint space without compromising potential publication in their target journal. To address this issue, the Transpose database was launched in 2019 to provide more clarity on journal policies regarding preprinting peer review, co-reviewing, and preprint policies relating to media coverage, licensing, versions, citation and platforms (this database is no longer actively maintained, perhaps reflecting the fact that journal policies are now less divergent than previously).

The success of *bioRxiv* meant that many publishers had to consider the degree to which the new culture of preprinting might threaten journal publishing or, conversely, how they could synergise with this ecosystem. Such discussions led to two major initiatives in the biological sciences – the launch of Review Commons, and the Preprint Review trial at *eLife* – which laid the groundwork for *eLife*’s current publishing model (see next section). Review Commons, launched by the European Molecular Biology Organization (EMBO) in collaboration with ASAPbio, provides researchers with the option of receiving journal-independent, high-quality peer review of their preprints and/or manuscript prior to submission to a journal (Lemberger and Pulverer, 2019). The result of the Review Commons process would be a ‘Refereed Preprint’ comprising the manuscript, reviewer reports and any author responses to those reports. Initially, public posting of the refereed preprint was optional, but Review Commons now requires authors to post manuscripts as preprints first (see EMBO news post), and the peer reviews and author response are posted on the preprint server by default (as detailed in a Review Commons news post). What has remained the same since the launch of Review Commons is that the authors can choose to submit their work, along with the reviews and responses, to one of the affiliate journals, which use these evaluations to make informed decisions without restarting peer review (Lemberger and Pulverer, 2019). Of note, the journals of The Company of Biologists were among the initial 17 affiliate journals – a group that has now grown to 28.

In response (and adding to) the evolving publishing landscape, two impactful papers appeared in *PLOS Biology* in 2019 that proposed extensive changes to the way scientific research is disseminated (Stern and O'Shea, 2019; Sever et al., 2019a,b preprint). In one of these, Bodo M. Stern and Erin K. O'Shea proposed a ‘publish first, curate second’ approach to academic publishing (Stern and O'Shea, 2019). This approach – perhaps better known as the ‘Publish, Review, Curate’ (PRC) model of publishing – aims to separate the dissemination and curation of scientific work. Importantly, it puts the authors in the driver's seat by allowing them to decide when and what to publish (Stern and O'Shea, 2019). Peer review then follows publication, thereby preventing the delay in dissemination. In line with this kind of thinking, another paper, which appeared at the same time, argues for funder preprint mandates (Sever et al., 2019a,b preprint). This plan, proposed by the co-founders of *bioRxiv* and the co-founder of PLOS, was termed Plan U (for ‘universal’) and aims to ensure accessibility of scientific literature and to support the implementation of new peer review and research evaluation initiatives, like the PRC model (Sever et al., 2019a,b preprint). These papers, and their proposals, have been hugely influential in further shaping the preprint and wider publishing ecosystem.

The outbreak of the COVID-19 pandemic at the end of 2019 further emphasised the importance of preprints for rapid information dissemination. The pandemic drove a surge in preprints, with 32% of COVID-19 papers listed on the National Institutes of Health (NIH)’s portfolio being preprints (as reported by ASAPbio). During this time, PubMed started a pilot experiment, as part of which they indexed preprints of NIH-funded authors (Funk et al., 2024 preprint; more information has also been provided by the National Library of Medicine).

Various preprint initiatives were launched during the COVID-19 pandemic, including *Rapid Reviews: COVID-19*, which received the 2022 PROSE Award for Innovation in Journal Publishing. It later evolved into a true overlay journal: *Rapid Reviews\Infectious Diseases*. Both Early Evidence Base (EEB) and Sciety were launched in 2020 as platforms focused on aggregating peer-reviewed preprints. As winner of the ASAPbio PreprintSprint, the main goal of EEB was to ensure that both expert evaluations and author responses are openly available, enabling readers to assess the findings critically (see EMBO news post). Similarly, the goal of Sciety (part of *eLife*) is to support preprint peer-review communities to openly share their efforts, allowing multiple groups, rather than a single journal, to participate in the review and curation of scientific literature.

## Increasing preprint visibility, funder adoption and the ‘Reviewed Preprint’

Following the pandemic, the adoption and visibility of preprints within the biological sciences has been steadily growing. In 2021, *bioRxiv* introduced a dashboard providing links to scientific discussion and evaluation of *bioRxiv* preprints (see *bioRxiv* news post). preLights, which had further developed into an invaluable resource by this time, with >1000 posts by early 2021, was fully integrated with the *bioRxiv* dashboard, featuring under the Community Reviews tab (see preLights news post). Not only through preLights did The Company of Biologists help to increase the reach and visibility of preprints, however: the journal Development introduced ‘In preprints’ articles highlighting key preprints in the field, sparking discussions and guiding readers to significant new research (Briscoe and Grewal, 2022).

In 2022, EMBO announced that refereed preprints would be recognised as an eligibility criterion for the EMBO Postdoctoral Fellowships. This move reflected a wider acceptance of preprints. Other funders had already started to include preprints in the evaluation of applicants, such as the Australian Research Council and European Research Council (see examples of funding agencies with changes to policies surrounding using preprints). In fact, some funders, such as Alex's Lemonade Stand Foundation, started to mandate the posting of preprints (see policy document). In March 2024, the Bill & Melinda Gates Foundation announced a future policy requiring all grantees to share their research as preprints – another big step toward the normalisation of preprints in scientific communication.

In 2023, the proportion of life sciences research disseminated as preprints grew to 10.7%, underscoring their sustained adoption (Levchenko et al., 2024). In response, *eLife* made a bold move in switching altogether to a model in which they would only publish ‘Reviewed Preprints’ (Eisen et al., 2022; see *eLife* news post). They created quite some ripples in the publishing ecosystem, and the impact of this drastic change of policy is still being evaluated (Behrens et al., 2024).

## Preprint revolution or evolution?

Preprints are here to stay, and The Company of Biologists remains determined to be at the forefront of this ever-growing movement. At Biology Open, the vision is to accelerate the dissemination of biological research by requiring that all journal submissions be posted as preprints (Adhikari et al., 2025). Platforms like preLights are more relevant than ever, offering curated and community-driven insights into the rapidly growing body of preprinted literature. Still, it's clear that preprinting and preprint peer review haven't (yet) replaced traditional journal publishing. The stranglehold of journal metrics and reputation still mean that researchers want and need formal publications for career progression. But also, more positively, there is recognition that journals provide important services to the community – not only in coordinating expert peer review, but also in helping to ensure ethical integrity and in collating and disseminating research results in an accessible format. Thus, preprints and journal articles complement each other – the one providing rapid access to research results, the other ensuring the long-term integrity and preservation of the scientific record. Moving forwards, we at The Company of Biologists will continue to experiment with our preprint-related activities and policies – in line with the ever-evolving needs of our communities.
